# Changes in Growth and Metabolic Profile of *Scutellaria baicalensis* Georgi in Response to Sodium Chloride

**DOI:** 10.3390/biology13121058

**Published:** 2024-12-17

**Authors:** Sylwester Ślusarczyk, Kajetan Grzelka, Joanna Jaśpińska, Anna Pawlikowska-Bartosz, Łukasz Pecio, Marta Stafiniak, Mehdi Rahimmalek, Wojciech Słupski, Adam Cieślak, Adam Matkowski

**Affiliations:** 1 Department of Pharmaceutical Biology and Biotechnology, Division Pharmaceutical Biology and Botany, Wroclaw Medical University, Borowska 211A, 50-556 Wroclaw, Poland; kajetan.grzelka@student.umw.edu.pl (K.G.); marta.stafiniak@umw.edu.pl (M.S.); 2 Laboratory of Experimental Plant Cultivation, Botanical Garden of Medicinal Plants, Wroclaw Medical University, Al. Jana Kochanowskiego 14, 50-367 Wroclaw, Poland; joanna.jaspinska@umw.edu.pl (J.J.); anna.pawlikowska-bartosz@umw.edu.pl (A.P.-B.); 3 Department of Biochemistry and Crop Quality, Institute of Soil Science and Plant Cultivation—State Research Institute, Czartoryskich 8, 24-100 Pulawy, Poland; lpecio@iung.pulawy.pl; 4 Department of Horticulture, College of Agriculture, Isfahan University of Technology, Isfahan 84156-83111, Iran; 5 Department of Food Chemistry and Biocatalysis, Wroclaw University of Life Sciences, ul. Norwida 1, 50-375 Wroclaw, Poland; 6 Department of Pharmacology, Wroclaw Medical University, 50-367 Wroclaw, Poland; wojciech.slupski@umw.edu.pl; 7 Department of Animal Nutrition, Poznan University of Life Sciences, Wolynska 33, 60-637 Poznan, Poland; adam.cieslak@up.poznan.pl

**Keywords:** sodium chloride, Baikal skullcap, baicalein, flavone glucuronides, bioactive compounds, amino acids

## Abstract

*Scutellaria baicalensis* Georgi is a valuable medicinal plant of the *Lamiaceae* family. Its roots have been used in Traditional Chinese Medicine (under the name Huang-qin) since antiquity and are nowadays included in Chinese and European Pharmacopoeias. It is abundant in bioactive compounds which constitute up to 20% of dried root mass. These substances are lipophilic flavones with unsubstituted B-ring, baicalein, and wogonin and their respective glucuronides–baicalin and wogonoside being the most abundant. The content of these compounds is variable and the environmental factors causing this remain partially unknown. The role of these compounds in stress response is still being investigated and in our efforts to measure the effect of NaCl treatment on *S. baicalensis* growth and metabolic profile, we hope to contribute to this research. Short-term exposure to salt stress (50, 100, and 150 mM NaCl) resulted in a marked increase of baicalein from 1.55 mg to 2.55 mg/g DM (1.6-fold), baicalin from 8.2 mg to 14.7 mg (1.8-fold), wogonin from 4.9 to 6.8 (1.4-fold), and wogonoside from 3.3 to 6.8 mg/g DM (2-fold) in the roots. Conversely, in the aerial parts, the content of individual major flavonoids: carthamidine-7-O-glucuronide and scutellarein-7-O-glucuronide decreased the most by 10–50% from 18.6 mg to 11.3 mg/g (1.6-fold less) and from 6.5 mg to 3.4 mg/g DM (0.52-fold less), respectively. The amino acid profile was also altered with an increase in root concentrations of the following amino acids: arginine from 0.19 to 0.33 mg/g (1.7-fold), glutamate from 0.09 to 0.16 mg/g DM (1.6-fold), alanine from 0.009 to 0.06 mg/g (6.8-fold), proline from 0.011 to 0.029 (2.4-fold) and lysine from 0.016 to 0.063 mg/g (3.9-fold). Aspartate concentration decreased from 0.01 to 0.002 mg/g (4.8-fold less) at 150 mM NaCl. In the aerial parts, the concentration and variation in levels of specific amino acids differed among groups. For instance, the glutamate content exhibited a significant increase exclusively in the treatment group, rising from 0.031 to 0.034 mg/g, representing a 1.2-fold increase. Proline concentration showed a marked increase across all treated groups with the highest from 0.011 to 0.11 mg/g (10-fold). In conclusion, moderate salt stress was shown to increase *S. baicalensis* root biomass and flavonoid content which is rarely observed in a glycophyte species and provides a foundation for further studies on the mechanisms of osmotic stress adaptation on the specialized metabolism level.

## Simple Summary

Salt challenges many cultivated plants by altering their growth, development, and composition. However, little is known about the influence of salt on medicinal plants that normally grow in low-salinity soils. *Scutellaria baicalensis* Georgi (Baikal skullcap) is an important medicinal plant rich in beneficial flavones. In this paper, we report the influence of NaCl addition to soil on the composition of these flavones in green parts and in the roots. It turned out that moderate doses of salt do not harm the plants and even increase the content of certain root constituents in favor of the most pharmacologically active. At the same time, we observed some alterations in the amino acid compositions which indicate physiological processes of adaptation of the plants to the stress related to excessive salinity.

## 1. Introduction

Salinity can negatively affect farm crops and industrially relevant plants, with a profound example of medicinal and aromatic plants, the quality of which depends on their metabolic profile. The fine-tuned specialized metabolism, e.g., biosynthesis of small molecular weight compounds that often possess pharmacological activity is considered one of the adaptive mechanisms in a plant’s response to stress [1,2,3].

*Scutellaria baicalensis* Georgi is a perennial from the *Lamiaceae* family. The roots, *Scutellariae baicalensis radix*, have been used for centuries in Traditional Chinese Medicine (TCM) under the name Huang-qin, either as a sole medicament or in multi-herbal preparations in the treatment of hepatitis, jaundice, cardiovascular diseases, diarrhea, insomnia, and hemorrhages as well as both bacterial and viral infections [4,5]. The raw material is most often harvested in spring and autumn [6].

Although it originates from the Asian Far East, nowadays it is widely grown in plantations worldwide to meet the ever-increasing demand. *S. baicalensis* has been successfully acclimated to cultivation in many regions outside of Asia, and the crop quality is comparable to that of the native range [7]. The herbal drug, *S. baicalensis radix*, has been recently included in both European and Chinese Pharmacopoeias, contributing to its growing popularity and importance, causing further demand for high-quality material. *S. baicalensis* roots are rich in flavonoids, however among over 40 such compounds the main bioactive components are lipophilic flavones with unsubstituted B-ring, present in the crude extract in the form of glucuronides–baicalin and wogonoside. Their aglycones–baicalein and wogonin, respectively—have been considered more pharmacologically active, but their content in the roots is much lower [5,6,7,8].

In several countries, including Poland, extracts from *Scutellariae baicalensis radix* are utilized in periodontal diseases, both the prevention and treatment [9,10]. The main compounds of *S. baicalensis* root extract were shown to improve liver function in various ailments [11] and possess potent anti-inflammatory [5], anticancer [12,13], antiradical, and antioxidant [14] properties, among many others. Baicalin is known for its wide spectrum of pharmacological activity and is considered a promising multi-therapeutic agent [15]. Baicalein—the most abundant aglycone—could be used in memory loss treatment, neurodegenerative disorders, mast cell-mediated asthma, and cancer [8,16,17,18]. Additionally, wogonin—the second major aglycone found in the *S. baicalensis* root—is not only a GABA-A receptor BDZ binding site ligand but also both MAO-A and MAO-B inhibitor and thus has great potential in depression and similar disorders treatment [4,19].

*S. baicalensis* plants grown in Europe have similar flavonoid content to those grown in Asia [13,20]. However, many factors such as stress stimuli influence the content and composition of major flavones in the roots. Many abiotic stressors, including UV radiation, drought, and high salinity, induce a metabolic response in plants that results in the enhanced production of harmful reactive oxygen species (ROS) [3]. Plants can be categorized based on their response to salt stress: halophytes, which are tolerant to high salinity and have developed various adaptation mechanisms, and glycophytes, which are not adapted to saline environments and thrive in low-salinity soils, such as the object of this study—*S. baicalensis*—for which salt exposure is an abiotic stressor. Most glycophytes experience reduced growth or even death when exposed to high salinity because they cannot effectively manage salt stress. Salt toxicity disrupts cellular processes, osmotic stress reduces water availability, and ion imbalance affects nutrient uptake [21]. Most crops, such as rice (*Oryza sativa* L.) [22], wheat (*Triticum aestivum* L.) [23], and beans (*Phaseolus vulgaris* L.) [24], as well as many plants of the moderate climate zones and forest species, are glycophytes.

It was observed that the accumulation of anthocyanin and other flavonoids in response to salt stress in DTC (hybrid) maize increased [25]. This could serve as a phytochemical strategy to combat toxic oxidative reactions subsequent to salt stress [26]. Other authors proved that anthocyanin in rice contributes to cellular protection by detoxifying accumulated salts [27]. Flavonoids may not only counteract oxidative stress but also prevent its occurrence. They are able to absorb high-energy quanta, preventing the development of photooxidative stress. The effect of UV light on flavonoid content in barley leaves was tested, and results revealed significant increases in flavonoid (saponarin, lutonarin) content when leaves were exposed to UV-B radiation, as compared to control conditions (absence of UV-B) [28]. Drought stress mitigation by flavonoids and their derivatives have been confirmed in *Arabidopsis thaliana* [29]. In our recent work, a pulsed electric field (PEF) was utilized, which also contributes to the generation of ROS in treated plants [30], we managed to promote *S. baicalensis* plant growth and significantly increase the content of major flavonoids in the root, especially the aglycones–baicalein and wogonin. However, it was accomplished only under specific treatment conditions [31].

The influence of the elevated salinity in the soil on the flavonoid content and profile in roots and aerial parts of *S. baicalensis* has not been reported so far. Hence, we have hypothesized that moderate concentrations of sodium chloride (NaCl) in the cultivation substrate would elicit an adaptive response in the phytochemical profile and may influence the growth performance of the plants belonging to an important medicinal species distinguished by an extraordinarily high content of flavones. Therefore, in this study, the content of major compounds including flavones in free and glycosidic forms was measured under various NaCl concentrations using HPLC, and the amino acid composition of the plant material was additionally analyzed to illustrate the complex response on a chemical level.

It was demonstrated that *S. baicalensis* reacted to the NaCl treatment with increased root biomass and a markedly altered phytochemical profile, both in terms of concentrations of compounds and free flavonoid to glucuronide form ratios.

## 2. Materials and Methods

### 2.1. Chemicals and Standards

All solvents used for this work (methanol, acetonitrile, n-hexane, dichloromethane, and ethyl acetate) were of analytical grade and purchased from Merck (Darmstadt, Germany). Ultrapure water was obtained from Milli-Q^®^ Simplicity 185 (Millipore Corp., Burlington, MA, USA) system. MS-grade formic acid (FA), as well as Baicalein (CAS Number 491-67-8), Baicalin (CAS Number 21967-41-9), Wogonin (CAS Number 632-85-9), Wogonoside (CAS Number 51059-44-0), Oroxylin A (CAS Number 480-11-5), Oroxylin A 7-glucuronide (CAS Number 36948-76-2), were purchased from Sigma-Aldrich (St. Louis, MO, USA).

### 2.2. NaCl Experiment

A completely randomized block experiment was carried out in a greenhouse as recommended in the literature with a fixed arrangement during the experiment [32]. The experimental plants were grown from seeds obtained from the mother plants that were part of the collection of the Botanical Garden of Medicinal Plants at the Wroclaw Medical University. Seeds were surface sterilized in 3% (*w*/*v*) NaOCl (sodium hypochlorite) for 5 min, then rinsed four times with sterilized distilled water and sown on Petri dishes containing wet tissue paper. Seeds were stratified at 4 °C for 4 days and placed vertically in a growth chamber at 22 °C, with a 16/8 h light/dark photoperiod. When the primary root length was approximately 2 cm, seedlings were transferred to the 500 cm^3^ volume pots filled with sphagnum peat substrate of pH 5.5–6.0 (one plant per pot). The experiment was conducted from February until July, in a detached greenhouse at the Institute of Soil Science and Plant Cultivation—State Research Institute (Department of Ecology and Soil Tillage in Wroclaw). The temperature inside ranged from 18 to 25 °C during the day and from 12 to 15 °C at night. Plants were grown in pots containing soil enriched with the following nutrients: phosphorus 35.5 mg, potassium 16.5 mg, and magnesium 2.2 mg per 100 g of soil, pH was 7.6. The pots were additionally fertilized with P + K+ Mg mixture (1.0 + 1.5 + 0.5 g, respectively) before sowing the seeds and with 3.6 g N 4 weeks after. At the beginning of flowering, 5-month-old plants were treated with NaCl to provoke a response to high salinity. For these experiments, a randomized block design in 4 groups was applied (control, 50, 100, and 150 mM NaCl) containing 6 plants each.

**Figure 1:**
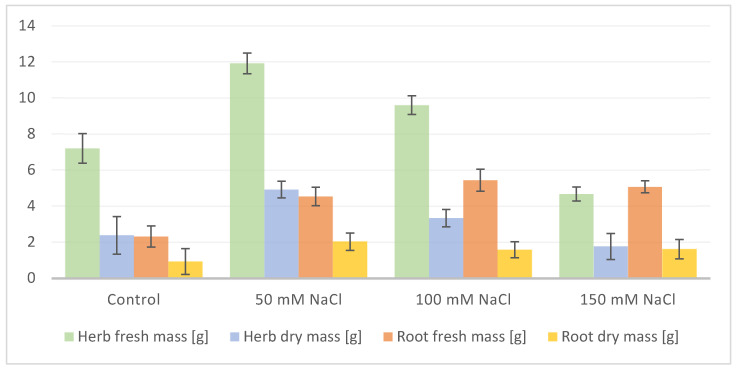
Average fresh and dry mass [g/plant] of *S. baicalensis* aerial (stems, leaves, flowers) and underground (roots) parts depending on NaCl concentration applied during watering. Control plants were watered with 0 NaCl. The error bars indicate standard deviation (±SD).

**Figure 2:**
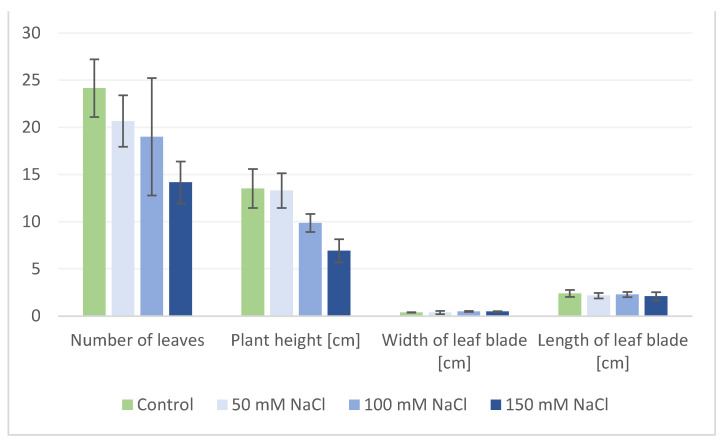
Average number of leaves, plant height, width, and length of the leaf blades of *S. baicalensis* under various NaCl concentrations. Control plants were watered with 0 NaCl. Error bars indicate standard deviation (±SD).

**Figure 3:**
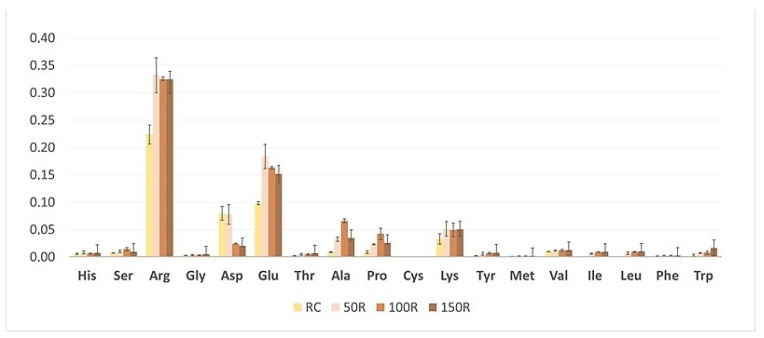
Average amino acid (AA) content in *S. baicalensis* root [mg/g dry mass]. RC—control plants watered with 0 NaCl, and 50R, 100R, and 150R—plants treated with 50, 100, and 150 mM NaCl solution, respectively. Error bars indicate standard deviation (±SD).

**Figure 4:**
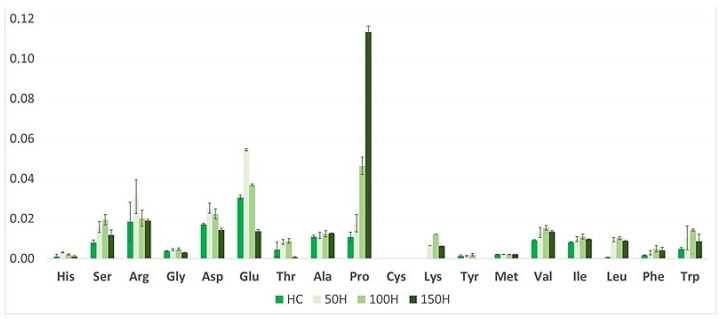
Average amino acid (AA) content in *S. baicalensis* aerial parts [mg/g dry mass]. HC—control plants watered with 0 NaCl, and 50H, 100H, and 150H—plants treated with 50, 100, and 150 mM NaCl solution, respectively. Error bars indicate standard deviation (±SD).

When plants started flowering, NaCl was applied to each pot (aside from control) every 2 days; firstly 50 mM in 50 mL of 50, 100, and 150 mM NaCl, respectively. A total of 50 mL of pure water acquired via reverse osmosis was applied to the control group and every pot was then treated with salt the next day. Afterward, the roots were separated and washed in water. Material (roots and aerial parts: leaves, stem and flowers) was weighed and frozen in liquid nitrogen and stored at −20 °C before freeze-drying and extraction. Biometric measurements including fresh biomass (noted 12 weeks after planting) of aerial parts and roots, plant height, number of leaves, length, and width of leaf blades (Figure 1 and Figure 2), and amino acid content (Figure 3 and Figure 4), as well as spectrometric analysis (Appendix A, Table A1 and Table A2), and statistical were conducted.

### 2.3. LC-MS Analysis

Right before freeze-drying (Christ Gamma 1–16 LSC, Martin Christ, Osterode am Harz, Germany), enzyme activity was stopped by immediately immersing the freshly harvested material in liquid nitrogen, and subsequently ground with a mortar. For future analysis, 50 mg of lyophilized powdered material from roots and 50 mg of aerial parts separately were suspended in 1000 µL of 80% methanol. After mixing for 1 min, the samples were sonicated in the ultrasonic bath (Intersonic, Olsztyn, Poland) with a pulse program set to 20 s of delivering ultrasounds and 5 s pause at 25 °C for 30 min. Afterward, the samples were centrifuged (23,000× *g*, for 5 min), and the supernatants were diluted 1:4 (with 0.1% formic acid in MeOH) before transferring them into the autosampler vials for LC-MS analysis. The *S. baicalensis* extracts were analyzed using a Dionex UltiMate 3000RS (Thermo Scientific, Darmstadt, Germany) system interfaced with a high-resolution quadrupole time-of-flight mass spectrometer (HR/Q-TOF/MS, Impact II, Bruker Daltonik GmbH, Bremen, Germany). The samples were separated on an Acquity UPLC BEH C18 column (100 × 2.1 mm, 1.7 μm, Waters, Manchester, UK) maintained at 30 °C. The mobile phase consisted of: A (0.1% formic acid in Milli-Q water, *v*/*v*) and B (0.1% formic acid in acetonitrile, *v*/*v*) at a flow rate of 0.4 mL/min. The gradient elution was: 2% B from 0 to 1 min with a short 0.3 min calibration segment, and the concentration of B was then increased to 60% from 1 to 20 min. The column was eluted with this concentration of solvent B for 4 min and then re-equilibrated for 0.3 min and back to 2% of B for the next 3.7 min. The samples were kept at 15 °C in the autosampler. The injection volume was 5.0 μL. The mass spectrometer operated in the positive and negative electrospray ionization ESI mode. The ion source operated with the following parameters: capillary voltage of 4.0 kV, corona voltage of 8.0 kV, nebulizer pressure of 2.5 bar, dry gas flow of 1.5 L/min, dry temperature of 200 °C, and vaporizer temperature of 320 °C. The mass scan range was from 50 to 1200 *m*/*z* with a 5 Hz spectral acquisition rate. MS/MS spectra were acquired in a data-dependent manner, whereby precursor ions (maximum 2) from each scan were subjected to collision-induced fragmentation if their absolute intensity exceeded 1800 counts. Variable collision energy ranging from 15 to 35 eV was used depending on the *m*/*z* of the selected precursor ion. The system was internally calibrated with sodium formate introduced to the ion source via a 20 μL loop at the beginning of each analysis using a six-port valve. Data were collected and processed by DataAnalysis 4.3 (Bruker Daltonik GmbH, Bremen, Germany). All analyses were performed in triplicates.

Standard solutions: baicalein, baicalin, wogonin, wogonoside, oroxylin A, and oroxylin A 7-glucuronide (Sigma-Aldrich, St. Louis, MO, USA) were prepared by dissolution in 80% (*v*/*v*) MeOH resulting in concentrations for each standard: 1.1, 0.9, 1.4, 1.2, 1.2 and 1.1 mg/mL. Such concentrations were then used to prepare the calibration curves based on seven concentration points (from 800 to 14 µg/mL) and a UV chromatogram was recorded at 280 nm. Obtained MS spectra were automatically exported to a ProfileAnalysis (Bruker, Daltonik GmbH, Bremen, Germany) spreadsheet. The retention time range was set between 2 and 22 min and the mass range was between 100 and 800 *m*/*z*. Rectangular bucketing with parameters acquired from time alignment was used. Data were normalized by the sum of bucket values in analysis and Pareto scaling. The value count of buckets was set to be equal to or greater than 25%. Then, multivariate analyses were performed—PCA and OPLS-DA using SIMCA-P 16 (Umetrics, Umea, Sweden). The significance of differences in concentrations of compounds was assessed by one-way ANOVA with Dunnett’s multiple comparison test (95% probability level) using GraphPad Prism version 9.

### 2.4. UPLC-MS/MS Amino Acids Analysis

Samples and standard solutions were injected into the UPLC system in duplicate. The UPLC^TM^ system (Waters ACQUITY) was equipped with a PDA detector in combination with a TQ detector (triple quadrupole mass spectrometer). The quantification method, including chromatographic conditions and UPLC-PDA-ESI-MS/MS system setup, was based on the previously described method [29]. The amount of particular amino acids was expressed in µg/g of dry plant material.

### 2.5. Amino Acid Content

The content of amino acids was analyzed using a derivatization approach based on available literature [33]. A total of 100 mg of the ground and lyophilized material from root and aerial parts were extracted with 50% (*v*/*v*) acetonitrile: water with the addition of 0.1% formic acid. Samples were sonicated in a water bath for 15 min and centrifuged for 5 min. The supernatants were pooled, and the cycle was repeated twice. Supernatants were combined and stored at −20 °C until analysis. Acetonitrile extracts were prepared in triplicate from each sample. Amino acid derivatization with AccQ·Tag Ultra Derivatization Kit (Waters Corp., Milford, MA, USA) was conducted according to the manufacturer’s protocol. Briefly, 10 µL of either a standard amino acid mix solution or a sample extract was mixed with 70 µL of AccQ·Tag Ultra borate buffer, and 20 µL of AccQ·Tag reagent previously dissolved in 1.0 mL of AccQ·Tag Ultra reagent diluent were added. The reaction was allowed to proceed for 10 min at 55 °C.

### 2.6. Statistical Analysis

The results were statistically analyzed utilizing the one-way ANOVA, set up in the form of randomized blocks in comparison to control using GraphPad Prism v. 9. The significance of differences was determined based on Dunett’s multiple comparison test.

## 3. Results

### 3.1. Phytochemical Profile of S. baicalensis Aerial and Underground Parts

The main biologically active compounds in *Scutellaria* genus are baicalein, scutellarein, and wogonin, as well as their glucuronides, baicalin, scutellarin, and wogonoside (Appendix A, Table A1). Main compounds of root extract identified were: baicalein (**59**), wogonin (**61**), baicalin (**43**), oroxylin A (**64**), apigenin (**57**), 5,6-dihydroxy-6-methoxyflavone (**58**), 5,7-dihydroxy-6,8-dimethoxyflavone-7-O-β-D-glucuronide (**53**), wogonoside (**52**), oroxylin A 7-O-β-D-glucuronide (**49**), 5,6,7-Trihydroxy-8-methoxyflavone-7-O-β-D-glucuronide (**48**), kaempferide 3-glucuronide (**35**), 8-C-glucose-6-C-arabinose-chrysin (**33**), 6-C-glucose-8-C-arabinose-chrysin (**27**), dihydroapigenin 7-O-[β-D-apiosyl-(1→2)-β-D-glucoside] (**18**), taxifolin (**10**), taxifolin 3-glucopyranoside (**3**), and tryptophan (**1**).

The composition of the metabolites in above-ground parts of *S. baicalensis* is not fully characterized in the literature, so the identification of the detected metabolites was challenging. Some of them have not been confirmed by any publication, so not every compound from aerial parts was successfully identified (Appendix A, Table A2). The main compounds were: carthamidine-7-O-β-D-glucuronide (**16**), isocarthamidin-7-O-β-D-glucuronide (**17**), scutellarein-7-O-β-D-glucuronide (**19**), schaftoside (**10**), 6-hydroxyluteolin-7-O-β-D-glucuronide (**12**), 6-Methoxyluteolin-3′-glucoside (**20**), isoscutellarein-7-O-β-D-glucuronide (**22**), dihydrobaicalin (**32**), norwogonin-7-O-β-D-glucuronide (**24**), baicalin (**30**), 5,7,8-trihydroxy-6-methoxyflavone-7-O-β-D-glucuronide (**34**), 5,7,2′,6′-tetrahydroxyflavonol-7-O-β-D-glucuronide (**36**), chrysin-7-O-β-D-glucuronide (**40**), 5,7-dihydroxyflavanone-7-O-β-D-glucuronide (**42**), and pinocembrin (**49**).

### 3.2. Impact of Salt Stress on Plant Growth and Amino-Acid Content

The sodium chloride treatment significantly influenced the plant condition and growth and the increase in soil salinity was accompanied by a decrease in plant height, number of leaves, and fresh mass of the aerial parts (Figure 1 and Figure 2 and Supplementary Figures S1 and S2).

In our experiment, after the NaCl treatment, the most notable rise in root concentrations of the following amino acids was noted (Figure 3 and Figure 4; Appendix A, Table A3): arginine (1.7-fold) in 50 mM NaCl, glutamate (1.7-fold) in 100 mM NaCl, alanine (6.8-fold) in 100 mM NaCl, proline (2.4-fold) in 100 mM NaCl and lysine (3.9-fold) in 150 mM NaCl. On the other hand, aspartate concentration significantly decreased (by 4.7 times) in the roots of 150 mM NaCl-treated plants. In aerial parts, the change in levels of the same amino acids was different, e.g., the content of glutamate increased only in the 50 mM NaCl group (1.2-fold). An increase in concentration of proline was the highest in the aerial parts of all treated plants (up to 10.3-fold) in 150 mM NaCl. There were no significant differences in concentrations among other amino acids; however, levels of almost all of these compounds in both roots and aerial parts have slightly increased. The multivariate data analysis highlighted the dose-dependent impact of NaCl on the content of amino acids in *S. baicalensis* aerial parts, e.g., a significant increase in the content of proline, leucine, and tryptophan paired with a decrease in arginine, aspartate, and glutamate concentrations was observed after administration of 150 mM NaCl. We aim to expand on these findings and elucidate the influence of NaCl on the aerial parts quality and phytochemical profile in this important medicinal crop.

### 3.3. Impact of Salt Stress on Phytochemical Profile

**Figure A1:**
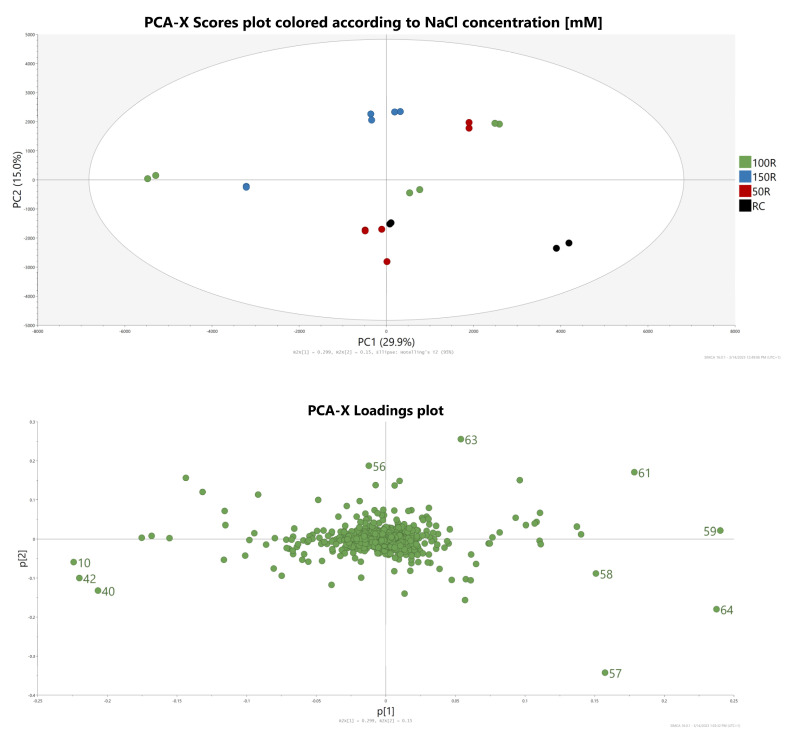
Principal component analysis (PCA) model presenting the relationship between the control (RC) and 50, 100, and 150 mM of NaCl treatment of *Scutellaria baicalensis* root (R) samples. The values used for loadings are found in Table 1. (PCA-X model, R^2^X_Cum_ = 0.863, Q^2^_Cum_ = 0.51).

**Figure A2:**
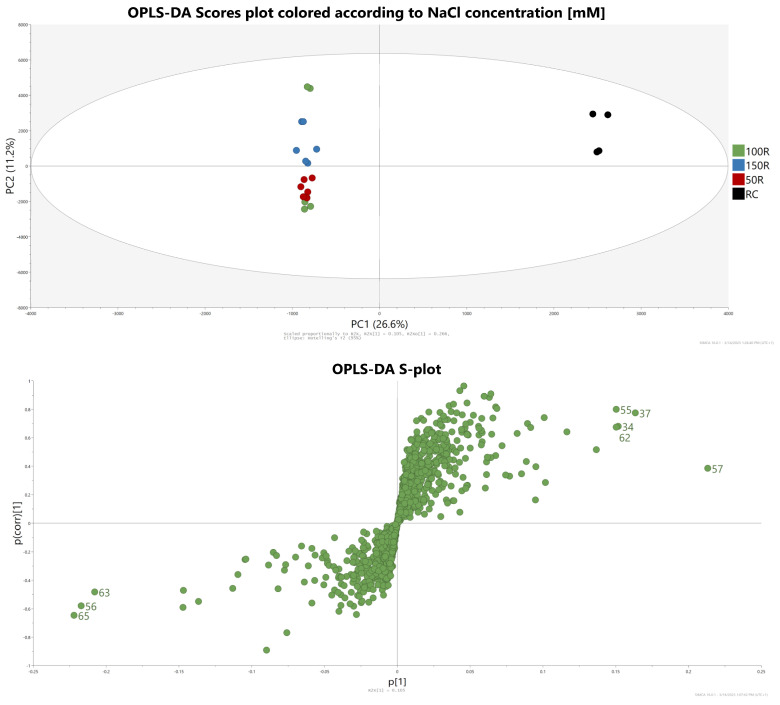
Orthogonal partial least squares—discriminant analysis (OPLS-DA) presenting the relationship between the control RC and 50, 100, and 150 mM of NaCl treatment of *Scutellaria baicalensis* root (R), and S-Plot of the OPLS-DA model for the control and treated groups. The axes on the S-plot from the predictive component are *p*1 vs. *p*(corr)1, representing the magnitude (modeled covariation) and reliability (modeled correlation), respectively. The numbers on the loadings plot correspond to compounds presented in Table 1. (OPLS-DA model: R^2^X_Cum_ = 0.635, R^2^Y_Cum_ = 0.999 Q^2^_Cum_ = 0.956).

**Figure A3:**
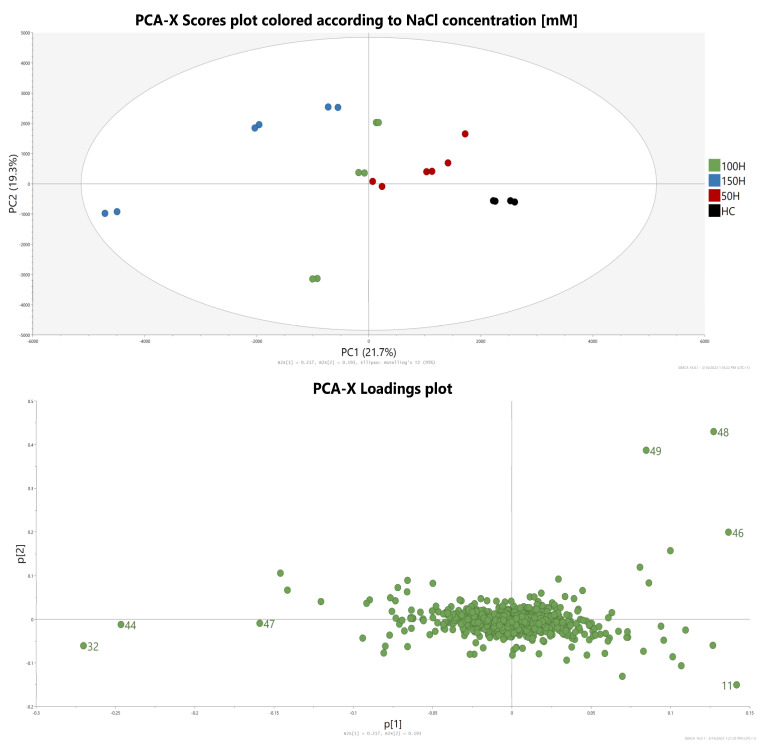
Principal component analysis (PCA) model presenting the relationship between the control HC and 50, 100, and 150 mM of NaCl treatment of *Scutellaria baicalensis* aerial parts. The values used for loadings are found in Table 2. (PCA-X model, R^2^X_Cum_ = 0.679, Q^2^_Cum_ = 0.334).

**Figure A4:**
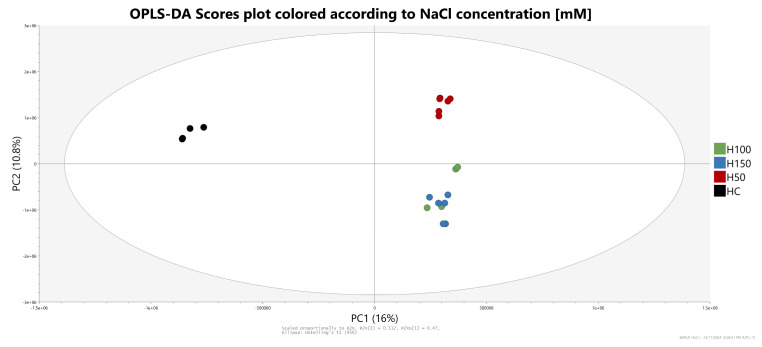

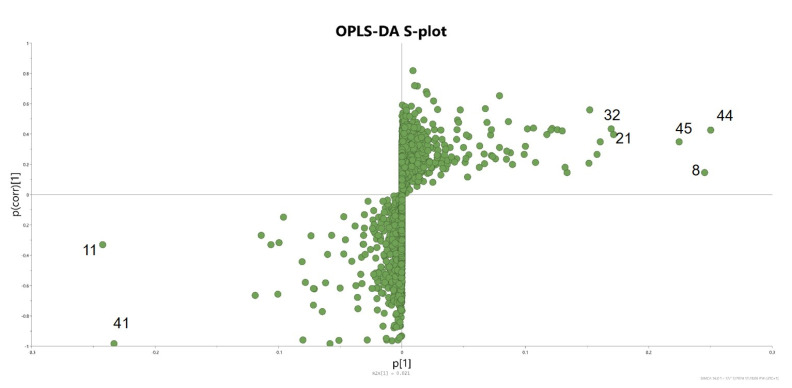
Orthogonal partial least squares—discriminant analysis (OPLS-DA) presenting the relationship between the control HC (0_NaCl) and (50, 100, 150 mM of NaCl) treatment of *Scutellaria baicalensis* aerial parts, and S-Plot of the OPLS-DA model for the control HC and 50, 100, 150 mM of NaCl. The axes on the S-plot from the predictive component are *p*1 vs. *p*(corr)1, representing the magnitude (modeled covariation) and reliability (modeled correlation), respectively. The values used for loadings are found in Table 2. (OPLS-DA model: R^2^X_Cum_ = 0.549, R^2^Y_Cum_ = 0.995 Q^2^_Cum_ = 0.941).

Baicalein (**59**) was the main metabolite in the *S. baicalensis* root profile after NaCl application. This flavone had the greatest impact on the entire multivariate analysis PCA model. We used a PCA score plot to present a natural correlation between the observations and to identify differential compounds. The OPLS-DA (Orthogonal Partial Least Squares Discriminant Analysis) model was used to explore differences in depth between profile metabolome of *S. baicalensis* depending on salt concentration. The OPLS-DA model with VIP values (VIP ≥ 1.0, |*p*(corr)| ≥ 0.5) was selected and the analysis results are shown in Appendix A; Figure A1 and Figure A2 where particular compounds with the most significant VIP are numbered according to Table A1 and Figure A3 and Figure A4 according to Table A2. Therefore, baicalein was also the main constituent that increased in response to salt stress. The two most abundant compounds in all root extracts were baicalein (**59**) and baicalin (**43**). An increase in their concentrations was highly evident after the application of 150 mM NaCl (Table 1) as their content changed from 15.5 mg to 25.5 mg (1.4-fold increase) and from 8.2 mg to 14.7 mg (1.8-fold increase) per g DM (dry mass) for (**59**) and (43), respectively. A rise in wogonin (**61**) and wogonoside (**52**) content was also noted under such conditions—from 4.9 to 6.8 (1.4-fold increase), and from 3.3 to 6.8 mg (2.1-fold increase) per g DM for (**61**) and (52), respectively. PCA loadings plot (Figure A1), highlights the significance of other compounds such as methyl hesperidin (**34**), dihydroapigenin 7-O-[β-D-apiosyl-(1→2)-β-D-glucoside] (**18**), oroxylin A (**64**), and 7-hydroxy-3-(2,4,5-trihydroxyphenyl)-3,4-dihydro-2H-1-benzopyran-4-one (**42**) in the presented model and, therefore, hints at their possible association with plant response to salt stress as well. However, under the same conditions, the concentrations of several compounds decreased, e.g., the content of oroxylin A (**64**) from 3.0 to 2.3 mg/g DM (1.3 times less). A decrease in root content of 5,7-dihydroxy-6,8-dimethoxyflavone-7-O-β-D-glucuronide (**53**) from 0.5 to 0.35 mg/g DM (1.4 times less), apigenin (**57**) from 1.3 to 0.4 mg/g DM (3.25 times less) and 5,6-Dihydroxy-6-methoxyflavone (**58**) from 1.2 to 0.7 mg/g DM (1.7 times less) was also observed. This suggests that under high salinity, *S. baicalensis* roots phytochemical profile shifted, resulting in an increase in main flavonoid content at the expense of compounds less involved in stress adaptation.

The constituents in the aerial parts of *S. baicalensis* were also identified in this study (Appendix A, Table A2). Under highest salinity (150 mM NaCl) the content of most aerial parts flavonoids decreased; carthamidin-7-O-β-D-glucuronide (**16**) from 18.6 mg to 11.3 mg/g DM (1.6 times less), isocarthamidin-7-O-β-D-glucuronide (**17**) from 5.8 mg to 1.4 mg/g DM (4.1 times less), scutellarein-7-O-β-D-glucuronide (**19**) from 6.5 mg to 3.4 mg/g DM (1.9 times less), 5,7,8-Trihydroxy-6-methoxyflavone-7-O-β-D-glucuronide (**34**) from 2.2 mg to 1.4 mg/g DM (1.5 times less). On the contrary, administering NaCl of the smallest concentration (50 mM) resulted in a steep increase in levels of scarcer compounds such as pinocembrin (**49**) from <LOQ to 0.18 mg/g DM, chrysin (**48**) from 0.01 to 41.21 mg/g DM (30-fold increase), 5,7-dihydroxyflavanone-7-O-β-D-glucuronide (**42**) from 0.03 to 1.55 mg/g DM (43-fold increase) and chrysin-7-O-β-D-glucuronide (**40**) from 0.39 to 3.65 mg/g DM (9.3-fold increase). On the S-Plot chart (Appendix A) pinocembrin (**49**) and isomer of 5,7,8,3′,4′-pentahydroxyflavanone-7-O-β-D-glucuronide (**11**) are marked as the most impactful on the proposed model, together with other relevant compounds such as **8**, **11**, **21**, **32**, **41**, **44** and **45**. These results confirm that salt stress affects the flavonoid content in aerial parts of *S. baicalensis* and that these parts contain mostly flavanones.

## 4. Discussion

Organ-specific localization of bioactive compounds was shown in *S. baicalensis* and *S. barbata* D. Don [34] where it was observed that baicalein, apigenin, wogonin, baicalin, norwogonoside, and wogonoside accumulated mainly in the roots of, while scutellarin was distributed in the aerial parts (stem, leaf, and flower). Other authors [35] point out that the distribution of flavonoids in *S. baicalensis* roots varies depending on their proximity to the stem, by which they can be divided into proximal, less fibrous “Ku Qin” and the distal “Tiao Qin”. Both are used in TCM, but the former is much richer in these flavones. In our study, LC-MS/MS analysis of the *S. baicalensis* metabolic profile resulted in the separation and identification of 65 substances in the roots and 49 in the aerial parts (Table 1 and Table 2, respectively). There are only a few reports [36,37] on the composition of the metabolites in the above-ground parts of *S. baicalensis*; therefore, the identification of the detected metabolites was challenging. Our results are in line with the previous findings and confirm the organ-specific accumulation of *S. baicalensis* flavonoids. The flavones lacking a 4′-OH group mainly accumulate in the root, while the aerial organs are much richer in flavanones for which salt exposure is an abiotic stressor. Glycophytes such as the object of this study—*S. baicalensis*—do not possess significant salt tolerance and instead develop complex resistance mechanisms [38]. One such response is the synthesis of highly hydrophilic compounds such as amino acids (glycine, proline, alanine, glutamine), amines (betaines), sugars, and polyols (mannitol, sorbitol, glycerol) or proteins (LEA proteins). These substances decrease the osmotic potential inside plant cells which prevents them from drying out and protects intracellular compounds susceptible to dehydration [39]. Proline is a major osmolyte which is also capable of scavenging ROS. Its levels were shown to rise under salt stress especially when the plant must make osmotic adjustments [25,40]. In our study, an increase in proline was observed both in roots and aerial parts but in the latter, its content surpassed all other amino acids at 150 mM NaCl treatment. Some other amino acids associated with osmotic adaptations also increased at 50 and 100 mM but mostly dropped back to the control level at 150 mM. It suggests that, similarly to many other plants, proline is used in *S. baicalensis* shoots as a major adaptive compound to cope with salt stress with some role of lysine and a branched amino acid—leucine. On the other hand, the substantial increase in arginine content in the roots may suggest alterations in the nitrogen metabolism and signaling, as arginine is involved in producing polyamines essential for plant stress responses [41]. The mechanisms behind the observed changes in aspartate and glutamate levels remain unclear, though. Hence, a diverged response between aboveground and underground parts is quite obvious in this species, providing an opportunity for further studies on this phenomenon.

These observations generally confirm that *S. baicalensis* produced more amino acids in response to high salinity but there are differences in distribution between roots and shoots, the latter being more sensitive [39,42]. The effect of abiotic stress on *S. baicalensis* was previously documented to have a strong negative impact on seed germination [43]. However, in the case of older plants, it was documented to influence the phytochemical content of the plant, especially regarding compounds from the flavonoid group [44].

According to the previous report [34], *S. baicalensis* seeds contain mainly flavonols during germination. As the plant grows, the major types of flavonoids shift to flavanones and flavones. A remarkable variation in aglycone/glucuronide ratio and content of baicalein/baicalin vs. wogonin/wogonoside was observed in different parts of the roots by Tani et al. [45], using a histochemical approach. The aglycones were mostly located in the outermost cortical layer as well as in the younger or thinner roots. Moreover, the wogonin-to-wogonoside ratio was higher in the pith. This corresponds to the observations we made (Table 1). This would be explainable if indeed the aglycones confer resistance to external stressors. The higher aglycone content in thin roots is also in agreement with our study where aglycones predominated even in untreated roots of the young plants. The salt stress treatment resulting in a further increase in aglycones at the expense of glucuronides would putatively be triggering the adaptive response to disrupted ionic balance in the young roots which are more actively participating in the uptake of mineral salts from the soil than the older ones [46]. However, under the salt stress conditions, changes in wogonin/wogonoside levels were less pronounced than those of baicalein/baicalin which suggests a more important role of baicalein in this process. Baicalein is an extremely efficient antioxidant whereas the less polar wogonin might play a more important role in other physiological functions, such as combating microbes [47]. In contrast, other researchers have found that the roots of 6-year-old plants contained large quantities of all flavones in the thickest, proximal sectors, and no differences in the proportions between them were noticed [35]. Older, fully mature plants would have the biosynthetic and accumulation mechanisms adjusted to the prolonged exposure to the environment, hence the stabilization of the metabolic profile during several years of growth in optimal conditions.

Biotic and abiotic stressors, such as high salinity can increase the accumulation of ROS in plants [3]. Flavonoids, as important non-enzymatic antioxidants, could enhance the stress tolerance of plants by scavenging ROS. This could be due to enzyme overexpression, such as R2R3-MYB proteins, mainly SbMYB8 which might have a major role in the regulation of flavonoid biosynthesis as well as improve drought and general plant stress tolerance [48,49]. In wheat, the flavonoid pathway gene expression and accumulation of these compounds may be closely related to drought tolerance and the flavonoid response mechanism may be different between cultivars [50]. A similar effect was also documented in *S. baicalensis*. A suitable level of drought stress can enhance baicalin accumulation by stimulating the expression and activity of the key enzymes involved in its biosynthesis. In this process, antioxidant enzymes are closely linked to the key enzymes of the secondary metabolic pathway, playing a crucial role in regulating the accumulation of baicalin [51]. It was also found that drought stress significantly affected the accumulation of four flavonoids and the expression of four related genes [52]. These findings may also be applicable in the case of salt stress, which similarly to drought leads to plant dehydration. Other studies indicated that the presence of phenolics, anthocyanins, and flavones is responsible for the increased salt tolerance of sugarcane [53]. The ability of *S. baicalensis* to alter the complex metabolic profile in response to exogenously applied stress factors was also studied by Shen et al. [54] who used sodium hydrosulfite spray to elicit oxidative stress. Similarly to our results, a significant upsurge of the aglycones (baicalein, wogonin, chrysin, 5,7,2′,6′-tetrahydroxyflavanon-3-ol) but not of glucuronides was noted. However, the content of both major flavones (baicalein, wogonin) increased in a similar manner, which differs from the results we obtained. However, it is worth noting that in the case of the cited study, 2-year-old plants were used, and the applied stressor was different. Na^+^ and Cl^−^ ions are considered the most important salt stress-inducing factors since Na^+^ in particular, causes deterioration of the soil’s physical structure and both Na^+^ and Cl^−^ are toxic to plants [38]. Chloride (Cl^−^) lowers the photosynthetic rate through inhibition of NO_3_-N uptake by the roots [55,56]. The level of NO_3_-N was significantly reduced in salt-stressed grape vines and this effect was correlated with decreased photosynthesis. The reduced NO_3_-N uptake combined with osmotic stress may explain the inhibitory effect of salinity on photosynthesis [57]. The effect of salinity on photosynthetic rate depends on salt concentration and plant species. There is evidence that low salt concentration may stimulate photosynthesis. For instance, in a salt water-tolerant mangrove plant, *Bruguiera parviflora* (Roxb.) [58], the photosynthetic rate increased at low and decreased at high salinity, whereas stomatal conductance was unchanged at low salinity and decreased at high salinity. After exposure to salt, the cell turgor decreased, and the stomata closed to conserve water. This can lead to less carbon fixation and the production of Reactive Oxygen Species (ROS) [59]. However, *S. baicalensis* is not a typical salt-tolerant species but a glycophyte, as it thrives naturally in the fertile soils of a vast area in East Asia. Even so, it is quite drought-resistant, and some data indicate better growth and quality under a slight water deficit [60]. Thus, our study showcases the potential of cultivating this valued herb in suboptimal conditions. The basis for this unexpectedly positive reaction to moderate salt stress can rely on the pleiotropic effect of the inherently high content of flavones. It is widely accepted that polyphenolic compounds such as flavonoids can efficiently neutralize free radicals not only as dietary phytochemicals but also in planta [61,62]. So, by increasing the salt stress level, the accumulation of flavonoids could be promoted to overcome the ROS; however, the flavonoids are more degradable in comparison with phenolic acids under severe stress conditions [63]. Moderate salinity stress can lead to the production of more flavonoids with less degradation but under stronger stress, the flavonoid content will decrease. Flavonoid biosynthesis is highly attributed to the availability of carbon, so the accumulation of flavonoids increases when carbon is in high supply [64]. Under severe stress, the equilibrium between sink and source becomes highly affected. To cope with this condition, the production of some polyphenolic compounds can be modulated by either up or down-regulation of the genes responsible for the production of these secondary metabolites [64].

Nonetheless, the lack of insight into the molecular regulation of the observed stress responses remains a limitation of the current study and future studies should verify the level of regulatory mechanisms by using differential transcriptomic and targeted manipulation on the genetic level (like, for example, gene editing or overexpression) to pinpoint the specific steps in the biosynthesis that are affected by salt stress. One of the candidates for the glucuronide/aglycone ratio would be the baicalein 7-O-glucuronosyltransferase but any other committed steps can also be considered both on transcriptional and posttranscriptional level [65]. The effect of stimulating growth and content of specialized metabolites in medicinal plants under moderate salt stress has been in effect widely described also in other species, such as *Ginkgo biloba* L. [66], a halophyte *Limonium bicolor* (Bag.) [67]. In *Glycyrrhiza uralensis* Fisch. [68], a 50 mM NaCl treatment caused a significant increase in both flavonoid and triterpene glycosides while aglycones were decreased or unchanged. This effect corroborated with higher transcription of most flavonoid pathway genes as well as in UDP-glycosyltransferases and MYB-class transcription factors. Hence, the mechanisms and reactions to salt treatment vary between species leading to diverse phytochemical profiles that can affect also their medicinal and nutritional properties.

In our study, the limitations include the potential influence of genetic diversity or other environmental factors that were not controlled during the experiment (temperature, humidity). However, all experimental plants were derived from a single population of free-pollinated plants cultivated in the Botanic Garden Collection for over 20 years [14]. Therefore, their genetic pool could be narrowed in comparison to plants from the indigenous populations in East Asia. On the other hand, *S. baicalensis* is cultivated in many other regions and little is known about its genetic diversity as a whole [69,70,71,72].

## 5. Conclusions

*S. baicalensis* reacted markedly to the salt content in the soil, depending on the NaCl concentration delivered with watering.

NaCl treatment affected growth and phytochemistry resulting in increased root and shoot biomass as well as flavone content in the roots.

The major flavonoids decreased in the shoots of NaCl-treated plants but also the aglycone-to-glucuronide ratio in the roots was highest at lower NaCl doses.

Therefore, introducing salt as a stressor may be considered as a means to increase both the plant biomass and content of the main quality markers of this medicinal crop, such as baicalein and baicalin. However, the salt level should be adjusted as if salinity levels are too high, the plant growth and metabolism are impeded. Furthermore, *S. baicalensis*, a species unique in its high and well-defined content of flavones, can be proposed as a feasible model for studying salt stress response pathways and regulatory mechanisms leading to complex modifications of the phytochemical profile.

## Figures and Tables

**Table 1:** The content of main flavonoids in *S. baicalensis* roots treated with different NaCl concentrations, analyzed using HPLC. Control plants were watered without added NaCl. (mg/g DM ± SD, *n* = 6).

Compound Name	Control	50 mM NaCl	100 mM NaCl	150 mM NaCl
Baicalein (**59**)	15.48 (±0.13)	22.38 (±0.10) ****	17.77 (±0.14) ****	25.49 (±0.12) ****
Baicalin (**43**)	8.23 (±0.06)	3.92 (±0.04) ****	3.27 (±0.04) ****	14.74 (±0.05) ****
aglycone/glucuronide ratio	1.9	5.7	5.4	1.7
Wogonin (**61**)	4.87 (±0.04)	4.83 (±0.04) ^ns^	4.17 (±0.05) ****	6.81 (±0.06) ****
Wogonoside (**52**)	3.31 (±0.06)	2.45 (±0.02) ***	2.23 (±0.03) ***	6.82 (±0.07) ****
aglycone/glucuronide ratio	1.5	2.0	1.9	1.0
Oroxylin A (**64**)	2.96 (±0.04)	2.00 (±0.02) ***	1.10 (±0.06) ****	2.29 (±0.04) ***
Oroxylin A glucuronide (**49**)	1.12 (±0.03)	0.90 (±0.02)	0.64 (±0.03)	2.13 (±0.04)
aglycone/glucuronide ratio	2.7	2.2	1.7	1.1
Apigenin (**57**)	1.29 (±0.05)	1.62 (±0.04) ***	0.32 (±0.02) ****	0.44 (±0.02) ****
5,7-Dihydroxy-6,8-dimethoxyflavone-7-O-glucuronide (**53**)	0.51 (±0.03)	0.29 (±0.001) ****	0.25 (±0.02) ****	0.35 (±0.01) ****
5,6-Dihydroxy-6-methoxyflavone (**58**)	1.18 (±0.04)	0.73 (±0.03) ****	0.86 (±0.02) ****	0.67 (±0.01) ****

Asterisks indicate the statistical significance of selected compound content calculated by one-way ANOVA test. Symbols correspond to statistically significant (**** *p* < 0.00005; *** *p* < 0.0005), ns—non significant.

**Table 2:** The content of main flavonoids in *S. baicalensis* aerial parts upon NaCl treatment, analyzed using HPLC. Control plants were watered without added NaCl. (mg/g DM ± SD, *n* = 6).

Compounds	Control	50 mM NaCl	100 mM NaCl	150 mM NaCl
Schaftoside (**10**)	0.55 (±0.02)	0.35 (±0.01) ****	0.33 (±0.02) ****	0.64 (±0.03) ***
6-hydroxyluteolin-7-O-glucuronide (**12**)	0.22 (±0.02)	0.03 (±0.0001) ****	0.38 (±0.01) ****	<LOQ
Carthamidine-7-O-glucuronide (**16**)	18.63 (±0.14)	13.86 (±0.09) ****	12.58 (±0.11) ****	11.39 (±0.07) ****
Isocarthamidin-7-O-*b*-D-glucuronide (**17**)	5.85 (±0.14)	8.04 (±0.11) ****	3.80 (±0.09) ****	1.44 (±0.08) ****
Scutellarein-7-O-glucuronide(**19**)	6.51 (±0.22)	2.72 (±0.17) ****	3.79 (±0.21) ****	3.35 (±0.18) ****
6-Methoxyluteolin-3′-glucoside (**20**)	0.10 (±0.002)	0.07 (±0.003) ****	0.05 (±0.002) ****	0.07 (±0.01) ***
Norwogonin-7-O-*b*-D-glucuronide (**24**)	0.74 (±0.15)	0.54 (±0.11) ^ns^	0.42 (±0.09) *	0.73 (±0.14) ^ns^
Baicalin (**30**)	<LOQ	<LOQ	<LOQ	<LOQ
Dihydrobaicalin (**32**)	0.02 (±0.00001)	0.18 (±0.04) ****	0.03 (±0.0002) ^ns^	<LOQ
5,7,8-Trihydroxy-6-methoxyflavone-7-O-*b*-D-glucuronide (**34**)	2.24 (±0.04)	1.20 (±0.08) ****	0.91 (±0.14) ****	1.40 (±0.08) ****
5,7,2′,6′-tetrahydroxyflavonol-7-O-*b*-D-glucuronoide (**36**)	0.18 (±0.03)	0.31 (±0.02) ***	0.19 (±0.009) ^ns^	<LOQ
Chrysin-7-O-b-D-glucuronide (**40**)	0.39 (±0.08)	3.65 (±0.41) ****	0.94 (±0.04) ****	0.11 (±0.02) ****
5,7-dihydroxyflavanone-7-O-*b*-D-glucuronoide (**42**)	0.04 (±0.004)	1.56 (±0.03) ****	0.20 (±0.01) ****	0.02 (±0.01) ^ns^
5,6,7-Trihydroxy-4′-methoxyflavone (**47**)	0.13 (±0.03)	0.05 (±0.0001) ****	0.05 (±0.0001) ****	0.30 (±0.05) ****
Chrysin (**48**)	0.01 (±0.003)	0.41 (±0.008) ****	0.20 (±0.004) ****	0.02 (±0.0001) ^ns^
Pinocembrin (**49**)	<LOQ	0.18 (±0.01)	0.04 (±0.01)	<LOQ
Baicalein (**46**)	0.25 (±0.01)	0.27 (±0.007) *	0.26 (±0.01) ^ns^	0.21 (±0.01) ***

Asterisks indicate the statistical significance of selected compound content calculated by one-way ANOVA test. Symbols correspond to statistically significant (**** *p* < 0.00005; *** *p* < 0.0005; * *p* < 0.05), ns-not significant.

## Data Availability

The experimental data are available upon request at sylwester.slusarczyk@umw.edu.pl.

## Supplementary Materials

The following supporting information can be downloaded at: https://www.mdpi.com/article/10.3390/biology13121058/s1, Figure S1. Roots after harvesting and washing showing difference in thickness between control plant (A) and plants treated with 100 mM NaCl (B)–thicker root. Figure S2. Overall view of plants treated with NaCl (H_2_O label means a control plant). The plant treated with 150 mM NaCl exhibits stress symptoms–less turgor and darker color in the leaves.

## Appendix A

**Table A1:** Main compounds from *Scutellaria baicalensis* root phytochemical profile. Identification based on mass spectrometer operated in the negative and positive electrospray ionization ESI mode compared with isolated standards ^a^ or tentatively identification with CompoundCrowler 3.1 program (Bruker Daltonoics Darmstad), supported with MetFrag database and appropriate literature.

No.	Compound [Reference]	Rt [min]	UV λ_max_ nm	*m*/*z* [M−H]^−^	Formula	MS^2^ Ion	*m*/*z* [M+H]^+^	MS^2^ Ion
1	tryptophan	1.8	220, 280	203.0000	C_11_H_12_N_2_O_2_	116 (100), 142 (47)	205.0971	188 (100), 146 (57)
2	unknown	2.6	220, 277	431.1561	C_19_H_28_O_11_	191 (100), 119, 299, 237	433.1704	455 (M-Na)
3	taxifolin-3-glucopyranoside	3.1	288	465.1049	C_21_H_22_O_12_	339 (100), 285 (74), 303 (12), 125 (100)		
4	taxifolin-7-glucopyranoside	4.6	290	465.1041	C_21_H_22_O_12_	303 (100)	467.1185	489 [M+Na], 305 (100), 287
5	quercetin-7-glucoside	5.0	253	463.0879	C_21_H_20_O_12_	301 (100), 285 (41), 177, 125, 151		
6	unknown	5.1		447.1501	C_19_H_28_O_12_	269 (100), 401 (24), 161, 121		
7	scuteamoenoside	5.2		463.1239	C_22_H_24_O_11_	301 (100), 139, 191, 161		
8	unknown rhamnosyl	5.4		475.1824	C_21_H_32_O_12_	113 (100), 149, 329 (54)	477.1962	
9	unknown	5.6		401.1451	C_18_H_26_O_10_	161 (100), 269 (75), 113 (28)		
10	taxifolin	6.9	210, 290	303.0510	C_15_H_12_O_7_	177 (100), 125 (58), 149 (24)	305.0653	
11	unknown	7.3		683.1476	C_29_H_32_O_19_	345 (100), 330 (41), 507 (11)		
12	5,7,3,2′6′-pentahydroxy flavanone	7.5	210, 290	303.0510	C_15_H_12_O_7_	177 (100), 125 (58), 149 (24)	305.0652	
13	luteolin 7-O-β-D-glucoside	7.9		447.0944	C_21_H_20_O_11_	285 (100)		
14	taxifolin-glucuronide	8.0		479.0838	C_21_H_20_O_13_	303 (100), 285 (17), 166 (40)		
15	salviaflaside	8.2		521.1308	C_24_H_26_O_13_	359 (100)		
16	apigenin 7-O-[β-D-apiosyl-(1→2)-β-D-glucoside]	8.3	220, 291, 335	563.1397	C_26_H_28_O_14_	239 (100), 209 (39), 443, 353, 269	565.1547	325 (100), 379 (89), 337 (84)
17	5,7,3,2′,6′-pentahydroxy flavanone-glucosyl (taxifolin 3-rhamnoside)	8.5	230, 291	449.1079	C_21_H_22_O_11_	125 (100), 177, 287	451.00	
18	dihydroapigenin 7-O-[β-D-apiosyl-(1→2)-β-D-glucoside]	8.7	220, 291, 335	565.1578	C_26_H_30_O_14_	239 (100), 209 (72), 269 (51)		
19	5,7,3,2′,6′-pentahydroxy flavone	8.75		301.0345	C_15_H_10_O_7_	149 (100), 151 (65), 175 (25), 125 (4)	303.0499	153 (100), 179 (50), 207 (48), 229, 137
20	6-C-glucose-8-C-rhamnose-chrysin	8.8	220, 273, 315	577.1552	C_27_H_30_O_14_	337 (100), 457 (75), 367 (59), 309 (13)	579.1709	309 (100), 279 (60), 363 (54), 333 (35)
21	eriodictyol-7-O-glucoside	9.2		449.1098	C_21_H_22_O_11_	287 (82), 161 (37), 125		
22	5,7,3,2′,6′-pentahydroxy flavanone (taxifolin)	9.3	210, 290	303.0510	C_15_H_12_O_7_	177 (100), 125 (58), 149 (24)	305.0653	
23	6-C-arabinose-8-C-glucose-glucose-chrysin	9.32		709.1960	C_32_H_38_O_18_	443 (100), 243	711.2122	549 (80), 363 (100), 375 (90), 393 (80), 321, 309
24	dihydroapigenin glucuronide	9.35		447.0938	C_21_H_20_O_11_	271 (100)		
25	dihydroscutellarein-7-O-β-D-glucuronide (eriodictyol 7-glucuronide)	9.4	215, 275	463.0895	C_21_H_20_O_12_	287 (100), 166 (30), 181 (15)	465.1024	289 (100), 271 (45), 169 (24)
26	scutellarein-7-O-β-D-glucuronide ^a^	9.5	220, 280, 330	461.0712	C_21_H_18_O_12_	285	463.0870	287
27	6-C-Glucose-8-C-arabinose-chrysin	9.6	215, 275, 315	547.1448	C_26_H_28_O_13_	337 (100), 367 (68), 427 (44), 457 (27)	549.1597	351 (100), 271 (35), 179, 167
28	5,7,2′-trihydroxy-6-methoxy flavone-7-O-β-D-glucuronide	9.7	215, 270, 337	475.0873	C_22_H_20_O_12_	284 (100), 299 (35), 285 (13.7), 300 (5), 165 (2.6)	477.1025	286 (100), 301 (73), 183 (6), 168 (5.5)
29	4′,5,7-trihydroxy- flavone (apigenin)-glucuronide-glucose	9.8	274	607.1323	C_27_H_28_O_16_	431 (100), 269 (42)		
30	eriodictin	9.85		433.1140	C_21_H_22_O_10_	167 (100), 197 (75), 287 (60)		
31	scutellarein-6,7-diglucuronide	9.9		637.1057	C_27_H_26_O18	285 (100), 461 (18)		
32	5,7,2′,5′-tetrahydroxy-8,6′-dimethoxyflavone-glucose	10.0	215, 264, 330	507.1158	C_23_H_24_O_13_	345 (100), 330 (80)		
33	8-C-glucose-6-C-arabinose-chrysin	10.1	215, 275, 315	547.1470	C_26_H_28_O_13_	167 (100), 197 (54), 287 (15)	549.1597	351 (100), 271 (35), 179, 167
34	methyl hesperidin	10.2		623.1993	C_29_H_36_O_15_	461 (100), 315 (13)		
35	kaempferide 3-glucuronide	11	208, 276, 329	475.0886	C_22_H_20_O_12_	299 (100), 284 (50)		
36	salviaflaside	11.2		521.1308	C_24_H_26_O_13_	359 (100)		
37	unknown	11.4		637.2119		461 (50), 175 (100), 315 (21), 160 (28)		
38	unknown	11.45		671.2935	C_32_H_48_O_15_	509 (100), 347 (25), 389 (18)		
39	hesperetin-7-O-β-D-glucuronide	11.5		477.1040	C_22_H_22_O_12_	301 (100), 286 (28), 181 (14)		
40	5,7,2′,5′-tetrahydroxy-8,6′-dimethoxyflavone	11.7	215, 265, 338	345.0607	C_17_H_14_O_8_	315 (100), 330 (15), 316 (14), 164 (6.5)	347.0759	289 (100), 314 (67), 317 (53), 169 (39), 150 (27)
41	{2-[5,7-dihydroxy-2-(4-hydroxyphenyl)-4-oxochromen-8-yl]-4,5-dihydroxy-6-(hydroxymethyl)oxan-3-yl}oxidanesulfonic acid	11.9		511.0557	C_21_H_20_O_13_S	269 (100), 431 (28)		
42	7-hydroxy-3-(2,4,5-trihydroxyphenyl)-3,4-dihydro-2H-1-benzopyran-4-one	12.3		287.0567	C_15_H_12_O_6_	125 (100), 161 (50), 201(16)		
43	baicalin ^a^	12.7	225, 280, 315	445.0771	C_21_H_18_O_11_	269 (100), 251 (8)	447.0922	271 (100), 253 (2)
44	dihydrobaicalin	12.8	215, 290	447.0929	C_21_H_20_O_11_	271 (100), 243 (58), 253 (13), 244 (97)	449.1076	273 (100), 169 (12), 123 (8), 131, 103
45	kaempferide diglucuronide	12.9	217, 285, 328	651.2297	C_30_H_36_O_16_	175 (100), 475 (11), 193 (21), 160 (18), 299		
46	5,7,-dihydroxy-6-methoxy flavone-7-O-β-D-glucoside	13.4	220, 280	445.1137	C_22_H_22_O_10_	267 (100), 283 (5)	447.0922	285 (100), 270 (45), 252(2)
47	norwogonin-7-O-β-D-glucuronide	13.7	215, 280, 360	445.0771	C_21_H_18_O_11_	269 (100)	447.0926	271 (100), 285 (3.8)
48	5,6,7-trihydroxy-8-methoxy flavone-7-O-β-D-glucuronide	13.8	215, 284	475.0879	C_22_H_20_O_12_	284 (100), 299 (16.7), 285 (13), 300, 283	477.1029	286 (100), 301 (97), 269
49	oroxylin A 7-O-β-D-glucuronide	13.9	210, 270, 310	459.0928	C_22_H_22_O_11_	268 (100), 283 (16), 269 (13)	461.1084	270 (100), 285 (73)
50	chrysin-glucuronide	14.0	270, 310	429.0821	C_21_H_18_O_10_	253 (100)	431.0975	255 (100)
51	5,7,8-trihydroxy-6-methoxy flavone-7-O-β-D-glucuronide	14.2	210, 290	475.0879	C_22_H_22_O_12_	284 (100), 299 (33), 285 (13), 300 (4.5)	477.1030	286 (100), 301 (88), 184 (4.3)
52	wogonoside ^a^ (wogonin-7-O-β-D-glucuronide)	14.4	220, 275, 345	459.0929	C_22_H_22_O_11_	268 (100), 283 (15), 269 (13), 284(2.2)	461.1085	270 (100), 285 (85)
53	5,7-dihydroxy-6,8-dimethoxyflavone-7-O-β-D-glucuronide	14.6	220, 280	489.1035	C_23_H_22_O_12_	298 (100), 283 (45), 299 (14), 313 (13)	491.1191	315 (100), 285 (85), 300 (36), 282 (28)
54	OH-norwogonin	14.8	215, 270	285.0413	C_15_H_10_O_6_	268 (100), 151 (15)		
55	5,7,4′-trihydroxy-6-methoxy flavone	15,0	217, 286	301.0727	C_16_H_14_O_6_	286 (100), 165 (74), 181 (24), 137 (17), 258 (8), 230 (4)		
56	5,2′,5′-trihydroxy-6,7,8-trimethoxyflavone	15.3	217, 285	359.0787	C_18_H_16_O_8_	329 (100), 344 (47), 314 (21), 194 (8)		
57	apigenin	15.6	220, 280	269.0453	C_15_H_10_O_5_	197 (100), 171 (40), 213 (31)	271.0604	169 (100), 139 (12), 123 (9), 141 (8)
58	5,6-dihydroxy-6-methoxyflavone	15.9	220, 280, 325	299.0561	C_16_H_12_O_6_	284 (100), 181 (14), 153 (14), 285 (13), 200 (11)	301.0709	286 (100), 184 (80), 156 (15), 137 (5), 169 (5)
59	baicalein ^a^	16.2	215, 275, 320	269.0457	C_15_H_10_O_5_	223 (24), 241 (20), 195 (17), 169 (15), 197 (12), 251 (11), 271 (8)	271.0603	123 (79), 253 (21), 169 (20), 103 (8)
60	skullcapflavone derivative	16.6	219, 275, 325	423.0397		343 (100), 328 (62), 313 (40), 298 (20), 269 (12)		
61	wogonin ^a^	19.0	210, 275	283.0616	C_16_H_12_O_5_	268 (100), 163 (23), 184, 239	285.0753	270 (100), 179 (8), 252 (7.2), 242, 168
62	chrysin	19.3	220, 270, 310	253.0510	C_15_H_10_O_4_	209	255.0648	153 (63), 129, 147, 152, 103
63	5,2′-dihydroxy-6,7,8,6′-tetramethoxyflavone (skullcapflavone II)	19.4	220, 270, 320	373.0934	C_19_H_18_O_8_	343 (100), 328 (64), 300 (24), 194 (10), 358	375.1075	345 (100), 197 (54), 327 (32), 169 (12), 227 (9)
64	oroxylin A ^a^	19.6	215, 270, 320	283.0613	C_16_H_12_O_5_	268 (100), 165 (8.8), 184 (7), 239	285.0754	270 (100), 168 (46), 140 (9), 242 (7.5), 224 (3)
65	4′,5-dihydroxy-6,7,8-trimethoxyflavone	20.3	220	343.0821	C_18_H_16_O_7_	313 (100), 298 (67), 270 (18.6), 299 (9), 328, 194	345.0966	315 (100), 197 (42), 297 (32), 169 (11), 287 (9)

**Table A2:** *Scutellaria baicalensis* aerial parts phytochemical profile. Identification based on mass spectrometer operated in the negative electrospray ionization ESI mode, compared with isolated standards ^a^, or tentatively identification carried out with the use of CompoundCrowler 3.1 program (Bruker Daltonics Darmstad), supported with MetFrag database and appropriate literature.

No.	Compound [Reference]	Rt (min)	UV λ_max_ nm	*m*/*z* [M−H]^−^	Formula	MS^2^ Ion
1	citric acid	1.0	220, 280	191.0196	C_6_H_8_O_7_	173 (54), 154 (15), 129 (8)
2	unknown	1.3	291	479.1045	C_21_H_20_O_13_	303 (100), 395 (74), 141 (51), 183 (15)
3	caffeic acid	4.8	320	179.0342	C_9_H_8_O_4_	135
4	kanokoside A	5.2		521.1878[M+FA]-	C_21_H_32_O_12_	329 (30), 311 (7), 161 (24)
5	1-O-Sinapoylglucose	6.2	281, 325	385.1139	C_19_H_28_O_11_	205 (100), 223 (41), 179 (15)
6	6-hydroxyluteolin	6.7	277, 310	301.0356	C_15_H_10_O_7_	283 (19), 178 (100), 211 (12)
7	apigenin-di-C-glucoside	7.7	275, 316	593.1509	C_27_H_30_O_15_	473 (100), 353 (74), 383 (24), 503 (21), 413 (8)
8	5,6,7,3′,4′-pentahydroxyflavanone-7-O-β-D-glucuronide [72]	7.9	286, 358	479.0839	C_21_H_20_O_13_	303 (100), 166 (27), 135 (13)
9	unknown	8.6	272, 334	625.1410	-	463 (100), 331, 168, 313, 287
10	schaftoside [72]	8.7	272, 334	563.1408	C_26_H_28_O_14_	473 (84), 353 (74), 383 (15)
11	isomer of 5,7,8,3′,4′-pentahydroxyflavanone-7-O-β-D-glucuronide	8.8	286, 358	479.0835	C_21_H_20_O_13_	303 (100), 166 (27), 135 (13)
12	6-hydroxyluteolin-7-O-β-D-glucuronide [72]	9.1	282, 342	477.1073	C_22_H_22_O_12_	301 (100), 283 (75), 273 (15)
13	taxifolin-7-O-β-D-glucoside [72]	9.2	210, 290	465.1045	C_28_H_18_O_7_	125 (100), 177 (74), 285 (15), 217, 339, 259
14	apigenin-6-C-Glu-8-C-Ara [72]	9.3	272, 336	563.1408	C_26_H_28_O_14_	473 (100), 443 (12), 413 (13), 383 (7), 353 (5)
15	isomer of taxifolin-7-O-β-D-glucoside	9.35	210, 300	465.1027	C_28_H_18_O_8_	303 (100), 259 (42), 285 (14)
16	carthamidin -7-O-β-D-glucuronide [72]	9.5	287, 365	463.0866	C_21_H_20_O_12_	287 (100), 166 (11), 269 (3), 181 (2), 193 (1), 175 (1)
17	isocarthamidin -7-O-β-D-glucuronide [72]	10.0	289, 341	463.0894	C_21_H_20_O_12_	287 (100), 166 (11), 269, 181, 175
18	isomer of isocarthamidin -7-O-β-D-glucuronide [72]	10.1	289, 341	463.0891	C_21_H_20_O_12_	287 (100), 166 (11), 269, 181, 175
19	scutellarein-7-O-β-D-glucuronide ^a^	10.2	220, 280, 330	461.0712	C_21_H_18_O_12_	285 (100), 267 (80), 161 (28)
20	6-methoxyluteolin-3′-glucoside	10.7	281, 333	477.1044	C_22_H_22_O_12_	315 (100), 393 (48), 191 (24), 261 (14)
21	unknown	10.9	326	623.1688	C_28_H_32_O_16_	447 (100), 429 (18), 315 (15), 193, 152
22	isoscutellarein-7-O-β-D-glucuronide [72]	11.1	220, 280, 330	461.0730	C_21_H_18_O_12_	285 (100)
23	naringenin-7-O-β-D-glucuronide [72]	11.2	211, 283, 334	447.0943	C_21_H_20_O_11_	271 (100), 151 (21), 177 (8)
24	norwogonin-7-O-β-D-glucuronide [30]	11.5	225, 280, 315	445.0779	C_21_H_18_O_11_	269 (100), 241 (52), 225 (18)
25	5,7,2′-trihydroxy-8-methoxyflavanone-7-O-β-D-glucuronoide [30]	11.6	282, 331	477.1039	C_22_H_22_O_12_	301 (100), 286 (37), 181 (14)
26	eugenol-O-glucuronide	11.7	314	339.1089	C_16_H_20_O_8_	163 (100), 148 (80)
27	unkown deoxyhexosyl	11.8	312	307.0830	C_15_H_16_O_7_	161 (100)
28	unkown acylglucosyl	12.5	274, 321	493.2293		451 (100), 289 (71), 161 (41), 221
29	scutellarein-5-O-glucuronide	12.6	214, 284, 335	461.0736	C_21_H_18_O_12_	285 (100), 267 (3)
30	baicalin [72]	12.7	225, 280, 315	445.0780	C_21_H_18_O_11_	269 (100)
31	scutellarein [30]	12.8	283, 331	285.0416	C_15_H_10_O_6_	239, 167
32	dihydrobaicalin [30]	12.9	283, 331	447.0940	C_21_H_20_O_11_	271 (100), 253 (5), 243 (2)
33	dihydroxyflavone glucuronide	13	283, 320	433.0789	C_20_H_18_O_11_	257
34	5,7,8-trihydroxy-6-methoxyflavone-7-O-β-D-glucuronide [30]	13.1	284, 332	475.0892	C_22_H_20_O_12_	299 (100), 284 (15)
35	unknown pentosyl	13.2	287, 345	447.2235	C_21_H_36_O_10_	315 (100)
36	5,7,2′,6′-tetrahydroxyflavonol-7-O-β-D-glucuronoide [30]	13.3	286, 338	477.1034	C_22_H_22_O_12_	301 (100), 273 (15), 283 (21)
37	baicalein 6-O-β-D-glucuronide [30]	13.4		445.0787		269 (100)
38	5,7,4′-trihydroxy-6-methoxyflavanone [30]	13.5	286, 362	301.0728	C_16_H_14_O_6_	165 (100), 286 (85), 181 (80), 165 (22), 119 (8)
39	norwogonin-8-O-β-D-glucuronide [30]	13.7	225, 280, 315	445.0784		269 (100), 225 (15), 197, 175
40	chrysin-7-O-β-D-glucuronide [30]	13.9	268, 311	429.0841	C_21_H_18_O_10_	253 (100), 209 (2), 143 (1)
41	5,7,4′-trihydroxy-8-methoxyflavanone [30]	14.2	286	301.0729	C_16_H_14_O_6_	286 (74), 165 (62), 181 (41), 195
42	5,7-dihydroxyflavanone-7-O-β-D-glucuronoide [30]	14.4	317	431.0996	C_21_H_20_O_10_	255 (100), 175 (2), 213
43	oroxylin A 7-O-β-D-glucuronide [72]	14.5	270, 310	459.0928	C_22_H_22_O_11_	268 (100), 283 (16), 269 (13)
44	trihydroxyflavone	15.0	289	271.0611	C_15_H_12_O_5_	151 (100), 177 (28), 227 (8), 119 (5)
45	apigenin [72]	15.6	267, 337	269.0463	C_15_H_10_O_5_	151 (52), 225 (8), 241 (8)
46	baicalein ^a^ [30]	16.2	215, 275, 320	269.0461	C_15_H_10_O_5_	151 (100), 241, 251, 195, 169
47	5,6,7-trihydroxy-4′-methoxyflavone [30]	16.6	217, 284, 334	299.0573	C_16_H_12_O_6_	284 (100), 165 (4)
48	chrysin [30]	19.2	212, 268	253.0513	C_15_H_10_O_3_	209 (100), 211 (41), 225 (8), 227
49	pinocembrin [30]	19.4	217, 289	255.0661	C_15_H_12_O_4_	213 (100), 151 (28)

**Table A3:** Average amino acids (AA) content (mg/g of DM) in *Scutellariae baicalensis radix* (R) and aerial parts (H), either untreated (C) or treated with 50, 100 and 150 mM NaCl. Standard deviation is presented in brackets. Asterisks indicate the statistical significance of AA content calculated by ANOVA test. Asterisks indicate the statistical significance of selected compound content calculated by ONE WAY ANOVA test (**** = *p* < 0.0001, *** = *p* < 0.001, ** = *p* < 0.05, * = *p* < 0.01, and a decrease in their number correlates to an increase in *p*-value). LOQ—limit of quantitation.

Amino Acids	HC	50H	100H	150H	RC	50R	100R	150R
HIS	0.001 (±0.001)	0.002 (±0.0002)	0.002 (±0.0003)	0.001 (±0.0005)	0.006 (±0.0012)	0.008 (±0.0025)	0.006 (±0.06)	0.73 (±0.14)
SER	0.008 (±0.001)	0.016 (±0.0028) **	0.019 (±0.0026) ****	0.011 (±0.0024)	0.007 (±0.04)	0.016 (±0.0022)	0.014 (±0.29)	0.98 (±0.25)
ARG	0.019 (±0.001)	0.031 (±0.0084) ****	0.02 (±0.0040)	0.019 (±0.0006)	0.20 (±1.72)	0.33 (±0.03) ****	0.32 (±0.36) ****	32.45 (±2.58) ****
GLY	0.004 (±0.002)	0.005 (±0.0005)	0.005 (±0.0005)	0.003 (±0.0002)	0.003 (±0.02)	0.004 (±0.0012)	0.003 (±0.03)	0.49 (±0.04)
ASP	0.017 (±0.005)	0.026 (0.0029) **	0.022 (±0.0025)	0.014 (±0.0007)	0.01 (±1.28)	0.091 (±0.02)	0.024 (±0.09) ****	2.01 (±0.12) ****
GLU	0.031 (±0.0012)	0.028 (±0.0077)	0.036 (±0.0005) *	0.014 (±0.0009) ****	0.01 (±0.29)	0.12 (±0.02) ****	0.16 (±0.21) ****	15.21 (±1.63) ****
THR	0.005 (±0.0036)	0.009 (±0.0014)	0.008 (±0.0011)	0.001 (±0.0004)	0.001 (±0.02)	0.007 (±0.0014)	0.008 (±0.08)	0.70 (±0.14)
ALA	0.011 (±0.0007)	0.011 (±0.0008)	0.012 (±0.0016)	0.012 (±0.0003)	0.009 (±0.07)	0.016 (±0.0036)	0.060 (±0.35) ****	1.82 (±0.43)
PRO	0.011 (±0.0023)	0018 (±0.0062) **	0.026 (±0.0043) ****	0.113 (±0.0030) ****	0.012 (±0.26)	0.023 (±0.0009)	0.030 (±1.05) *	2.58 (±0.21)
CYS	<LOQ	<LOQ	<LOQ	<LOQ	<LOQ	<LOQ	<LOQ	<LOQ
LYS	<LOQ	<LOQ	<LOQ	<LOQ	0.016 (±0.94)	0.024 (±0.011)	0.042 (±1.27) ***	6.37 (±1.24) ****
TYR	0.001 (±0.04)	0.001 (±0.0004)	0.002 (±0.006)	<LOQ	0.003 (±0.00)	0.56 (±0.0024)	0.007 (±0.12)	0.78 (±0.07)
MET	0.002 (±0.00)	0.002 (±0.00)	0.002 (±0.00)	0.002 (±0.00)	0.001 ± (0.01)	0.15 (±0.0004)	0.001 (±0.03)	0.16 (±0.01)
VAL	0.01 (±0.02)	0.013 (±0.0025)	0.016 (±0.001) *	0.013 (±0.000)	0.01 (±0.04)	0.012 (±0.0011)	0.012 (±0.15)	1.24 (±0.16)
ILE	0.008 (±0.01)	0.01 (±0.0013)	0.011 (±0.001)	0.009 (±0.0002)	0.001 (±0.01)	0.009 (±0.0011)	0.01 (±0.04)	0.96 (±0.06)
LEU	0.0006 (±0.01)	0.01 (±0.0011) ***	0.01 (±0.0007) ***	0.009 (±0.0001) **	0.05 (±0.01)	0.009 (±0.0020)	0.01 (±0.06)	1.02 (±0.04)
PHE	0.002 (±0.02)	0.003 (±0.0011)	0.49 (±0.001)	0.004 (±0.0015)	0.001 (±0.04)	0.002 (±0.0010)	0.002 (±0.10)	0.24 (±0.04)
TRP	0.003 (±0.07)	0.011 (±0.0049) **	1.43 (±0.0006) ****	0.009 (±0.0034)	0.003 (±0.20)	0.006 (±0.0009)	0.010 (±0.29)	1.65 (±1.46)
