# Supplementary material for: Changes in Growth and Metabolic Profile of Scutellaria baicalensis Georgi in Response to Sodium Chloride
Source: Biology (Basel). 2024 Dec 17;13(12):1058. doi: 10.3390/biology13121058 (PMC11673518; doi:10.3390/biology13121058)
Supplement: Supplementary file 1 [file biology-13-01058-s001.zip › Supplementary Figure 1S-2S.pdf]

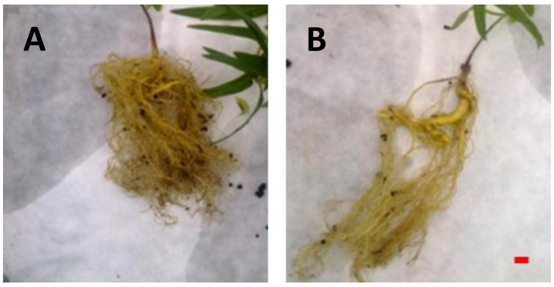

Supplementary Figure S1. Roots after harvesting and washing showing difference in thickness between control plant (A) and plants treated with 100 mM NaCl (B) – thicker root.

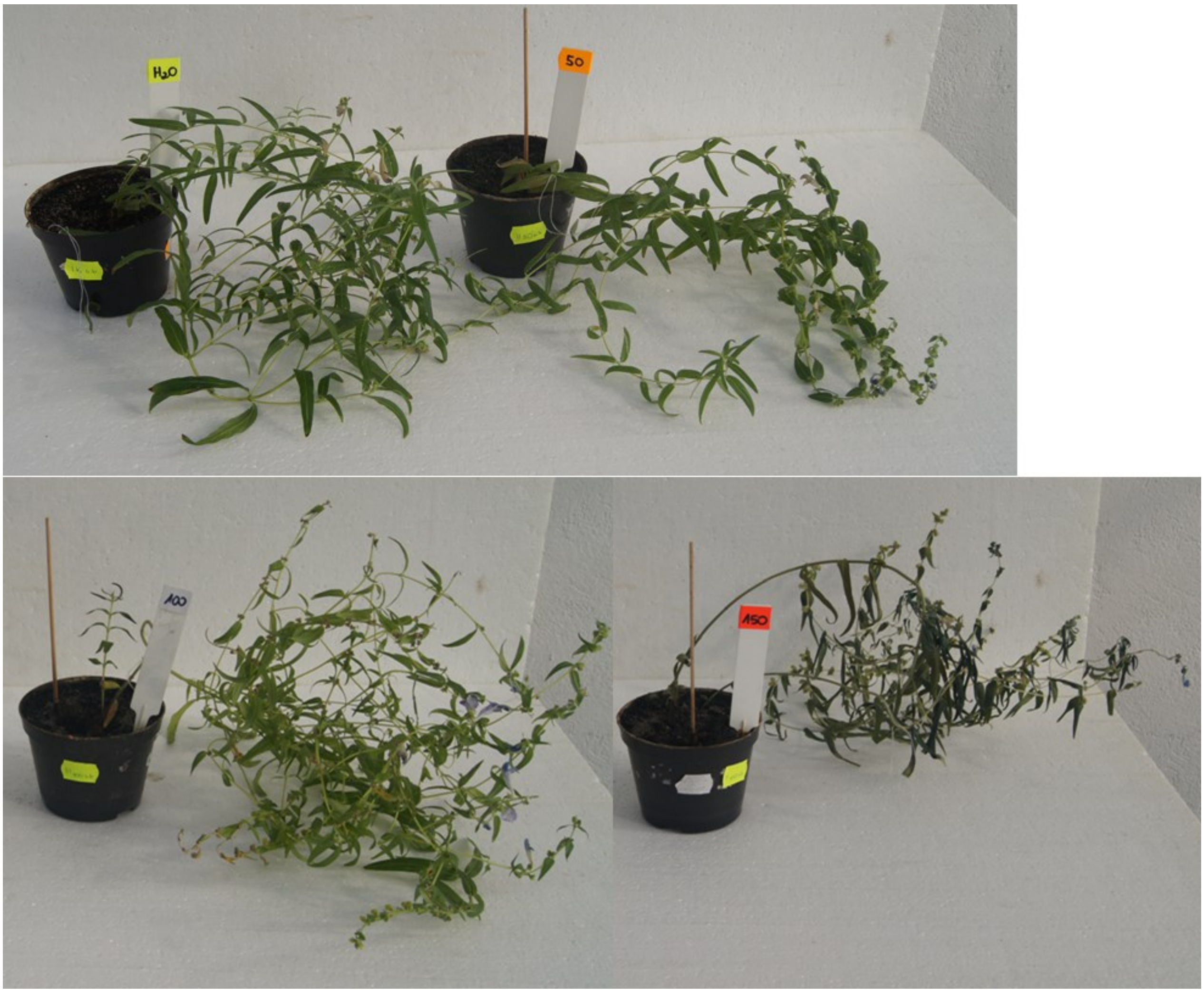

Supplementary Figure S2. Overall view of plants treated with NaCl (H_2O label means a control plant). The plant treated with 150 mM NaCl exhibits stress symptoms – less turgor and darker color in the leaves.
